# Prevalence of and risk factors associated with sexual health issues in primiparous women at 6 and 12 months postpartum; a longitudinal prospective cohort study (the MAMMI study)

**DOI:** 10.1186/s12884-018-1838-6

**Published:** 2018-05-31

**Authors:** Deirdre O’Malley, Agnes Higgins, Cecily Begley, Deirdre Daly, Valerie Smith

**Affiliations:** 1 Health Research Board, Research Fellow, School of Nursing and Midwifery, Trinity College Dublin, Dublin, Ireland; 2 School of Nursing and Midwifery, Trinity College Dublin, Dublin, Ireland; 3 Institute of Health and Care Sciences, The Sahlgrenska Academy, University of Gothenburg, Gothenburg, Sweden

**Keywords:** Sexual health postpartum, Prevalence, Dyspareunia, Sexual activity, Perineal trauma, Breastfeeding, Regression analysis

## Abstract

**Background:**

Many women are not prepared for changes to their sexual health after childbirth. The aim of this paper is to report on the prevalence of and the potential risk factors (pre-pregnancy dyspareunia, mode of birth, perineal trauma and breastfeeding) for sexual health issues (dyspareunia, lack of vaginal lubrication and a loss of interest in sexual activity) at 6 and 12 months postpartum.

**Methods:**

A longitudinal cohort study of 832 first-time mothers who were recruited in early pregnancy and returned postnatal surveys at 3, 6, 9 and 12 months postpartum were assessed for sexual health issues and associated risk factors.

**Results:**

Nearly half of the women (46.3%) reported a lack of interest in sexual activity, 43% experienced a lack of vaginal lubrication and 37.5% of included women had dyspareunia 6 months after birth. On univariate analysis, vacuum-assisted birth, 2nd degree perineal tears, 3rd degree perineal tears and episiotomy were all associated with dyspareunia 6 months postpartum, but, of these only 3rd degree tears, in association with breastfeeding and pre-existing dyspareunia, remained significant on multivariable analysis. Breastfeeding, in combination, with other significant factors, was associated with dyspareunia, a lack of vaginal lubrication and a loss of interest in sexual activity 6 months postpartum, and, dissatisfaction with body image emerged as a significant factor associated with lack of interest in sexual activity at 12 months postpartum. Pre-pregnancy dyspareunia and breastfeeding emerged as common factors associated with all three outcomes of dyspareunia, a lack of vaginal lubrication and a loss of interest in sexual activity at 6 months postpartum.

**Conclusion:**

Breastfeeding and pre-existing dyspareunia are associated with sexual health issues at 6 months postpartum. Pre-existing dyspareunia is associated with a lack of vaginal lubrication at 12 months postpartum and breastfeeding is associated with dissatisfaction with body image. Preparing women and their partners during the antenatal period and advising on simple measures, such as use of lubrication to avoid or minimise sexual health issues, could potentially remove stress, anxiety and fears regarding intimacy after birth. Introducing the topic of pre-existing sexual health issues antenatally may facilitate appropriate support, treatment or counselling for women.

## Background

Discourse on women’s sexual health after birth is gaining momentum across diverse disciplines, for example, midwifery, obstetric, sexology and psychology disciplines [1–5]. This increased interest and body of research in perinatal sexual health, however, is not evidenced in sexual health policy [6, 7] or maternity care policy [8, 9], although data demonstrating that women are not prepared for changes to their sexual health after birth [10], are available. Lack of knowledge and preparation for sexual health issues postpartum can be distressing for women, and their partner, while also negatively impacting on their ability to adapt to their new role as mothers [10–12]. Postpartum sexual health is challenging to theoretically define but cannot be separated from sexuality and sexual function, and is thought to be influenced by labour and birth events [13]. Attributes of good postpartum sexual health include; sexual desire, resumption of sexual intercourse after birth, pain free sex and orgasm. Several studies to date have focused on factors such as timing of resumption of sexual intercourse [4, 14] and frequency of sexual intercourse [15, 16] and are often limited to the first 3 to 6 months postpartum [17–20]. Others, in measuring women’s postpartum sexual health tend to do so with instruments not validated for use in a postpartum population; for example, the Female Sexual Function Index [21–23], the Arizona Sexual Experience Scale [24] and the Golombok Rust Inventory of Sexual Satisfaction [25]. Furthermore, health professionals themselves have identified a lack of expertise on advising women about potential changes to sexual health after birth [26]. Studying women’s sexual health for a lengthy period of time postpartum, for example, up to 1 year postpartum, from the perspectives of women themselves (i.e. self-report) is paramount so as to gain a deeper understanding of potential sexual health issues affecting women, insight into any issues that may persist or worsen over time and an understanding of factors that are associated with emergent issues. Gaining an understanding of issues can assist healthcare professionals plan healthcare practices or interventions to address these, and, in doing so, positively impact the sexual health of women who give birth.

The Maternal health And Maternal Morbidity in Ireland (MAMMI) study, launched in February 2012 (www.mammi.ie) is a longitudinal cohort study investigating the existence, extent and prevalence of an array of morbidities (mental health issues, sexual health issues, urinary incontinence, faecal incontinence, pelvic girdle pain, etc.) in nulliparous women antenatally and up to 1 year postpartum across three maternity units in Ireland. The survey was launched in the three maternity units on a rolling basis; February 2012 (site 1), September 2013 (site 2) and August 2015 (site 3). Data were collected via self-reported questionnaires in early pregnancy and at 3, 6, 9 and 12 months postpartum, and from hospital records, so that changes over time might be evaluated. Women received the study information, consent form and Survey 1 on their first visit (the booking visit) to the hospital. Those who completed and returned Survey 1 and the consent form were sent Surveys 2 to 5 by post with a stamped addressed envelope provided for return, at 3, 6, 9 and 12 months postpartum, respectively, unless they indicated, during this time, that they wished to withdraw from the study. At time of analysis, a total of 2764 women joined the study, representing 38% of all those who were invited to take part (*n* = 7348) and future plans involve following this cohort of women up to 5 years postpartum.

In this paper we report on the prevalence of sexual health issues (i.e., dyspareunia, lack of vaginal lubrication, a loss of interest in sexual activity) and the potential factors (pre-pregnancy dyspareunia, mode of birth, perineal trauma, and breastfeeding) that might be associated with these at 6 and 12 months postpartum in a cohort of 832 women from one study site (site 1) who completed all 5 MAMMI study surveys between February 2012 and July 2015. Limiting to this site was necessary as data collection and entry in sites 2 and 3 was ongoing at the time of the analysis and complete data were only available from site 1.

## Methods

### Study design

A longitudinal prospective cohort study was conducted, evaluating sexual health issues self-reported by women, at 6 and 12 months postpartum, recruited to the MAMMI study from one large urban maternity hospital in Ireland. Surveys, providing the study data, were returned between February 2012 and July 2015 (see http://mammi.ie/surveys.php for downloadable copies of the MAMMI surveys).

### Sample

Women were eligible to take part if they were nulliparous (no previous live birth or pregnancy ending in stillbirth), aged 18 years or over and had sufficient English to complete the surveys. No additional exclusion criteria were applied. Midwives and midwifery students offered eligible women the study invitation pack at women’s first antenatal appointment, which takes place usually between 12 and 16 weeks gestation, and all women who accepted the study information were telephoned within 1–2 weeks of their booking visit. The purpose of this call was to offer women additional information on the study, answer questions, and determine their interest in taking part. Women were regarded as recruited to the study when they returned the completed consent form and Survey 1.

### Ethical approval

Ethical approval for the study was granted by the Faculty of Health Sciences Research Ethics Committee, Trinity College Dublin and the Research Ethics Committee of the participating hospital study site.

#### Data collection and outcomes measures

The MAMMI study surveys are A4 booklets of approximately 60 pages in length, taking 40–50 min to complete. All surveys sought information on sexual health issues, within a discrete survey section, and women’s demographics (e.g. age, relationship status, employment status, highest level of education) were additionally collected in Survey 1. The surveys were developed from surveys used in a similar cohort study, the Maternal Health Study, in Melbourne, Australia [27], and were subsequently assessed for face validity (with 15 women), content validity (with 18 experts), tested for reliability using the test-retest method (with 11 women) (Cohen’s Kappa co-efficient 0.87 to 1.0), piloted (with a sample of 33 women) and modified accordingly for use in an Irish maternity population with permission from The Maternal Health study team. Specific information that related to sexual health morbidity centred on issues such as the occurrence of a lack of vaginal lubrication, dyspareunia (pain during sexual intercourse), difficulty in reaching orgasm, inability to reach orgasm, vaginal tightness, vaginal looseness and a loss of interest in sexual activity. In Survey 1 women were asked to report on these symptoms, if any, in the previous 12 months and since becoming pregnant. In the four postpartum surveys women were again asked to self-report on any of these issues for the 3 months preceding their 3, 6, 9 and 12 months postpartum time-frames.

Data on mode of birth, perineal trauma and birth events were collected from the hospital records using a detailed pre-designed data extraction form and in the first postpartum (3 month) survey. Mode of birth was classified into 5 categories; spontaneous vaginal birth, vacuum birth, forceps birth (including failed vacuum birth), emergency caesarean section (CS) (included failed forceps birth) and elective CS (includes elective CS in labour). Grades of perineal trauma were classified into 6 categories; intact perineum (includes women who had a CS), 1st degree tear (includes women who had both sutured and unsutured 1st degree tears), 2nd degree tears, episiotomy (includes women who had an extended episiotomy), 3rd degree tears and labial and vaginal wall lacerations. Breastfeeding at each time point was ascertained through one question *‘Are you still breastfeeding your baby or giving expressed breastmilk’?* Women were also asked to rate their satisfaction with their body image at each time point, indicating if they were *‘always satisfied’*, ‘*sometimes satisfied’* or ‘*never satisfied’* with their body image.

#### Statistical analysis

Data were analysed using IBM SPSS (version 23). Frequencies and descriptive statistics were used to present prevalence rates of sexual health issues in the year before this pregnancy, in early pregnancy and at 6 and 12 months postpartum. To determine if there was any statistically significant change over time in sexual health issues, McNemar’s Chi-squared test for differences in correlated proportions was calculated [28].

Univariate and multivariable logistic regression analyses, using Odds Ratio (OR) and 95% Confidence Intervals (CI) were used to assess associations between pre-pregnancy dyspareunia, mode of birth, perineal trauma and breastfeeding, and, dyspareunia, a lack of vaginal lubrication and a loss of interest in sexual activity at 6 and 12 months. These three sexual health issues were chosen for the analyses as they are the more commonly reported of all sexual health issues. The multivariable logistic regression analysis model included the variables; age, pre pregnancy body mass index (BMI) and level of education. The Omnibus Test of Model Coefficients and the Hosmer and Lemeshow Test supported the models used.

## Results

### Characteristics of the study participants

**Fig. 1:**
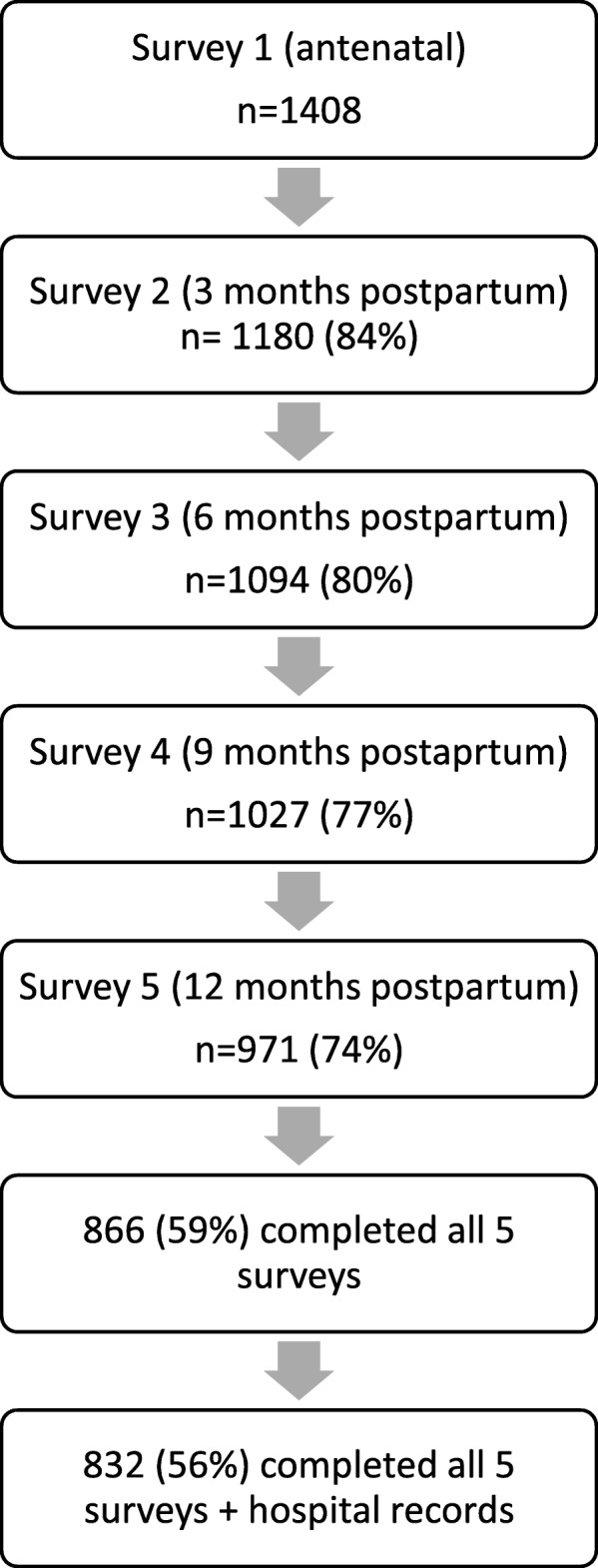
Analytical sample

Women included in this study report (*n* = 1477) all gave birth between August 2012 and end of July 2014. Of these 1477 women who were recruited in early pregnancy, 1408 were eligible for follow-up. For those 69 women not available for follow-up, reasons included, gave birth elsewhere, withdrew at Survey 1, experienced a late miscarriage or stillbirth, and no consent provided. Subsequent 2, 3, 4 and 5 survey return rates were 1180 (84%) 1094 (80%), 1027 (77%) and 971 (74%), respectively. To determine, accurately, any changes over time only data from women who returned all five surveys (*n* = 866, 59%) and consented to having their hospital records accessed (*n* = 832, 56%) were included in these analyses (Fig. 1).

Where it was possible to do so, study data were compared to data in the Irish National Perinatal Statistics Report for 2013 [29]. This allowed for an assessment of the national representativeness of the study participants. The National Perinatal Statistics Report is produced annually and collates data (hereafter referred to as national data) on the obstetric and social characteristics of every woman who gave birth in Ireland in the year preceding the report. Comparative assessments demonstrated that the study sample had proportionately fewer women under 24 years of age and more 30–34 and 35–39 year old women when compared to national statistics (30–39 years: 70.1% in the MAMMI study versus 52.5% in the national data). Greater than two-thirds (*n* = 566, 68.1%) of women in the MAMMI study are Irish with just over a quarter (*n* = 216, 25.9%) born in another European country. The five most common countries of birth after Ireland were; Poland (*n* = 58, 7%), United Kingdom (*n* = 45, 5.4%), France (*n* = 13, 1.6%), Germany (*n* = 12, 1.5%) and Romania (*n* = 12, 1.5%). Seventy-one percent of participants had a university degree or higher (*n* = 588, 70.6%). No data were available for this item from the National Perinatal Statistics Report; however, the Central Statistics Office reports a national rate of women aged 25–34 with a third level qualification in Ireland of 55.3% in 2014 [30].

Women in the MAMMI study were under represented in terms of spontaneous vaginal birth (35.6% versus 45.5% nationally), over represented for forceps births (12% versus 5.6% nationally) and representative for caesarean section rates (31.6% versus 27.2% nationally). One third (*n* = 301, 36.1%) of study participants had an episiotomy compared to nearly half (*n* = 1187, 46.3%) of nulliparous women at the hospital site in 2013. The study sample were representative in all other categories of perineal trauma compared to the research site. Table 1 presents the characteristics of the MAMMI study sample.

**Table 1:** Characteristics of study participants

Characteristics of study participants		Study participants	
		n	%
Age	Up to 24	41	4.9
	25 to 29	179	21.5
	30 to 34	376	45.2
	35 to 39	207	24.9
	40 and over	29	3.5
Place of birth	Irish	566	68.1
	Europe (excluding Ireland and UK)	171	20.5
	UK	45	5.4
	America	17	2
	Asia	10	1.2
	Africa	8	0.9
	Australia	3	0.4
	Missing	12	1.4
Highest level of education	School - second level	89	10.7
	Apprenticeship	75	9.1
	Certificate or Diploma	77	9.3
	Undergraduate degree	254	30.5
	Postgraduate degree	334	40.1
	Missing	3	0.3
Mode of birth	Spontaneous vaginal birth	296	35.6
	Vacuum birth	172	20.7
	Forceps birth	101	12
	Elective Caesarean Section	74	8.9
	Emergency Caesarean Section	189	22.7
Perineal trauma	Intact	268	32.2^a^
	1st degree tear	43	5.2
	2nd degree tear	168	20.2
	3rd degree tear	26	3.1
	Episiotomy	301	36.1
	Labial/vaginal wall tears	26	3.1

^a^includes participants who had a CS

### Prevalence of self-reported sexual health issues over time

**Fig. 2:**
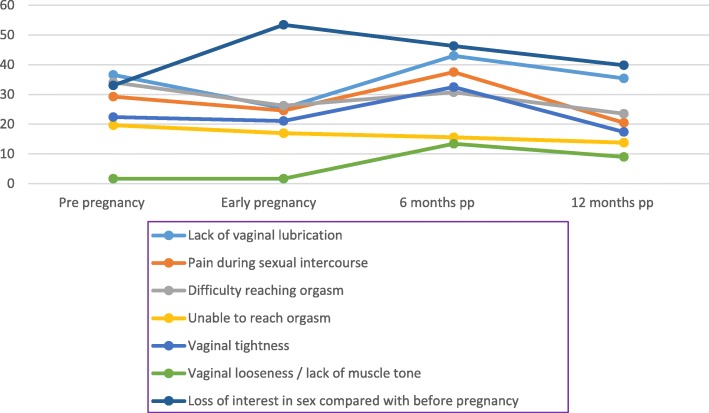
Self-reported sexual health issues; pre pregnancy, in early pregnancy, 6 and 12 months postpartum

Table 2 presents the number and proportion of women who experienced sexual health issues pre-pregnancy, in early pregnancy and at 6 and 12 months postpartum. The prevalence of loss of interest in sexual activity was considerably elevated 6 months postpartum (46.3%) and remained significantly so at 12 months postpartum compared to pre-pregnancy levels (39.8% versus 33% *p* < 0.001). The proportion of women reporting dyspareunia at 6 months was significantly higher than those who experienced it pre-pregnancy (37.5% versus 29.3%, *p* < 0.001). Contrastingly, this was significantly lower than pre-pregnancy levels at 12 months postpartum (20.5% versus 29.3% *p* < 0.001). Six months postpartum 43% of women reported a lack of vaginal lubrication compared to 36.6% pre-pregnancy (*p* = 0.002). This decreased to 35.4% 12 months after birth (*p* = 0.761). Pregnancy and birth appeared to resolve difficulties women experienced with orgasm, as, pre-pregnancy, 34.1% of women experienced difficulty achieving orgasm and 19.7% were unable to achieve orgasm. The prevalence of these sexual health issues were significantly less, however at 12 months after birth (23.5% (*p* < 0.001) and 13.8% (*p* = 0.001), respectively). Figure 2 illustrates the prevalence of sexual health issues experienced by women at the different time points.

**Table 2:** Prevalence of self-reported sexual health issues pre pregnancy, in early pregnancy and at 6 and 12 months postpartum

	Pre pregnancy n (%)	Early pregnancy n (%)	6 months pp. n (%)	12 months pp. n (%)
Lack of vaginal lubrication *m = 174*	241/658 (36.6)	167/658 (25.4)	283/658 (43)	233/658 (35.4)
Pain during sexual intercourse *m = 203*	184/629 (29.3)	155/629 (24.6)	236/629 (37.5)	129/629 (20.5)
Difficulty reaching orgasm *m = 236*	203/596 (34.1)	156/596 (26.2)	183/596 (30.7)	140/596 (23.5)
Unable to reach orgasm *m = 254*	114/578 (19.7)	98/578 (17)	90/578 (15.6)	80/578 (13.8)
Vaginal tightness *m = 216*	138/616 (22.4)	130/616 (21.1)	200/616 (32.5)	107/616 (17.4)
Vaginal looseness / lack of muscle tone *m = 243*	10/589 (1.7)	10/589 (1.7)	79/589 (13.4)	53/589 (9)
Loss of interest in sexual activity compared with before pregnancy *m = 187*	216/654 (33)	349/654 (53.4)	303/654 (46.3)	260/654 (39.8)

*m* missing responses; *pp*. postpartum

### Univariate logistic regression analysis

#### Mode of birth as a risk factor for postpartum sexual health issues

Six months postpartum, vacuum-assisted birth was significantly associated with dyspareunia (OR 1.6, 95% CI 1.1–2.4), elective CS was associated with a reduced odds of experiencing dyspareunia (OR 0.5, 95% CI 0.3–0.9), and an emergency CS was protective of experiencing a loss of interest in sexual activity 6 months postpartum; these associations did not persist to 12 months postpartum. There was no significant association between vacuum-assisted birth and an increased lack of vaginal lubrication at 6 (OR 1.3, 95% CI 0.9–2.0) and 12 months postpartum (OR 1.3, 95% CI 0.9–1.9) (Table 3).

**Table 3:** Mode of birth as a risk factor for sexual health issues at 6 and 12 months postpartum

	6 months postpartum					12 months postpartum				
	n/total	%	Unadjusted OR	95% CI	*p* value	n/total	%	Unadjusted OR	95% CI	*p* value
Dyspareunia										
Spontaneous vaginal birth	108/279	38.7	1.0 (ref.)			60/280	21.4	1.0 (ref.)		
Vacuum birth	83/163	50.9	1.6	1.1–2.4	0.013*	47/164	28.7	1.5	0.9–2.3	0.086
Forceps birth	30/89	33.7	0.8	0.5–1.3	0.397	18/96	18.8	0.8	0.4–1.5	0.577
Elective CS	16/66	24.2	0.5	0.3–0.9	0.03*	10/64	15.6	0.7	0.3–1.4	0.3
Emergency CS	61/181	33.7	0.8	0.5–1.2	0.277	31/181	17.1	0.8	0.5–1.2	0.258
Lack of vaginal lubrication										
Spontaneous vaginal birth	122/285	42.8	1.0 (ref.)			93/286	32.5	1.0 (ref.)		
Vacuum birth	83/166	50	1.3	0.9–2.0	0.139	63/164	33	1.3	0.9–1.9	0.206
Forceps birth	36/92	39.1	0.9	0.5–1.4	0.535	33/96	34.4	1.1	0.7–1.8	0.738
Elective CS	22/65	33.8	0.7	0.4–1.2	0.187	20/64	31.3	0.9	0.5–1.7	0.845
Emergency CS	77/181	42.5	1.0	0.7–1.4	0.955	69/185	37.3	1.2	0.8–1.8	0.287
Loss of interest in sexual activity										
Spontaneous vaginal birth	145/285	50.9	1.0 (ref.)			112/286	39.2	1.0 (ref.)		
Vacuum birth	88/165	53.3	1.1	0.7–1.6	0.615	69/165	41.8	1.1	0.8–1.6	0.579
Forceps birth	40/95	42.1	0.7	0.4–1.2	0.139	40/96	41.7	1.1	0.7–1.8	0.664
Elective CS	25/67	37.3	0.6	0.3–1.0	0.047	29/65	44.6	1.2	0.7–2.1	0.419
Emergency CS	73/181	40.3	0.6	0.4–0.9	0.027*	63/179	35.2	0.8	0.6–1.2	0.391

*indicates statistical signficance at *p* < 0.05

#### Perineal trauma as a risk factor for postpartum sexual health issues

Compared to women with an intact perineum, women who had 2nd degree perineal tears (OR 1.6, 95% CI 1.0–2.3), episiotomy (OR 1.7, 95% CI 1.2–2.5) or 3rd degree perineal tears (OR 3.7, 95% CI 1.5–9.3), were significantly more likely to experience dyspareunia at 6 months postpartum. This association persisted to 12 months for both episiotomies and 3rd degree perineal tears (Table 4). At 6 months postpartum a loss of interest in sexual activity was associated with both 2nd and 3rd degree perineal tears (Table 4).

**Table 4:** Perineal trauma as a risk factor for sexual health issues at 6 and 12 months postpartum

	6 months postpartum					12 months postpartum				
	n/total	%	Unadjusted OR	95% CI	*p* value	n/total	%	Unadjusted OR	95% CI	*p* value
Dyspareunia										
Intact	77/252	30.6	1.0 (ref.)			40/250	16	1.0 (ref.)		
1st degree tear	11/39	28.2	0.9	0.4–1.9	0.766	7/40	17.5	1.1	0.5–2.7	0.811
2nd degree tear	66/161	41	1.6	1.0–2.3	0.03*	35/158	22.2	1.5	0.9–2.5	0.119
3rd degree tear	13/21	61.9	3.7	1.5–9.3	0.005*	8/25	32	2.5	1.0–6.1	0.05*
Episiotomy	121/281	43.1	1.7	1.2–2.5	0.003*	69/288	24	1.6	1.1–2.6	0.023*
Labial or vaginal wall tear	10/24	41.7	1.6	0.7–3.8	0.267	7/24	29.2	2.1	0.8–5.6	0.109
Lack of vaginal lubrication										
Intact	101/251	40.2	1.0 (ref.)			91/254	35.8	1.0 (ref.)		
1st degree tear	16/39	41	1.0	0.5–2.1	0.926	14/42	33.3	0.89	0.5–1.8	0.754
2nd degree tear	72/163	44.2	1.2	0.8–1.8	0.428	57/162	35.2	1.0	0.6–1.5	0.894
3rd degree tear	12/23	52.2	1.6	0.7–3.8	0.269	9/24	37.5	1.0	0.4–2.6	0.87
Episiotomy	128/288	44.4	1.2	0.8–1.7	0.325	97/289	33.6	0.9	0.6–1.3	0.58
Labial or vaginal wall tear	11/25	44	1.2	0.5–2.7	0.715	10/24	41.7	1.3	0.5–3.0	0.57
Loss of interest in sexual activity										
Intact	103/253	40.7	1.0 (ref.)			94/249	37.8	1.0 (ref.)		
1st degree tear	17/41	41.5	1.0	0.5–2.0	0.928	19/42	45.2	1.4	0.7–2.6	0.358
2nd degree tear	88/163	54	1.7	1.1–2.5	0.008*	69/162	42.6	1.2	0.8–1.8	0.327
3rd degree tear	15/24	62.5	2.4	1.0–5.8	0.044*	9/25	36	0.9	0.4–2.2	0.863
Episiotomy	138/288	47.9	1.3	0.9–1.9	0.093	114/289	39.4	1.1	0.8–1.5	0.687
Labial or vaginal wall tear	10/24	41.7	1.0	0.4–2.4	0.927	8/24	33.3	0.8	0.3–2.0	0.67

*indicates statistical signficance at *p* < 0.05

#### Breastfeeding as a risk factor for postpartum sexual health issues

When data on women who were breastfeeding and not breastfeeding were compared, the results showed that women who were breastfeeding were significantly more likely to experience dyspareunia (OR 1.9, 95% CI 1.3–2.6), a lack of vaginal lubrication (OR 1.7, 95% CI 1.2–2.3) and a loss of interest in sexual activity (OR 1.7, 95% CI 1.3–2.3) 6 months postpartum. This association was not significant at 12 months postpartum, likely due, perhaps, to the low numbers still breastfeeding 12 months after birth. It is noteworthy that for those breastfeeding, the ORs for dyspareunia, a lack of vaginal lubrication and a loss of interest in sexual activity were all greater than 1.0 at 12 months postpartum, although none reached a level of significance (Table 5).

**Table 5:** Breastfeeding as a risk factor for sexual health issues at 6 and 12 months postpartum

	6 months postpartum					12 months postpartum				
	n/total	%	Unadjusted OR	95% CI	*p* value	n/total	%	Unadjusted OR	95% CI	*p* value
Dyspareunia	139/292	47.6	1.9	1.3–2.6	< 0.001*	33/133	24.8	1.2	0.7–1.8	0.477
Lack of vaginal lubrication	159/295	52.9	1.7	1.2–2.3	0.001*	55/135	40.7	1.3	0.9–1.9	0.221
Loss of interest in sexual activity	168/298	56.4	1.7	1.3–2.3	0.001*	62/135	45.9	1.4	1.0–2.1	0.064

*indicates statistical signficance at *p* < 0.05

#### Pre-pregnancy dyspareunia as a risk factor for postpartum sexual health issues

Women who reported pre-pregnancy dyspareunia were significantly more likely to report several postpartum sexual health issues including dyspareunia at 6 and 12 months postpartum, a lack of vaginal lubrication at 6 and 12 months and a loss of interest in sexual activity at 6 and 12 months postpartum compared to those who did not report it (Table 6).

**Table 6:** Pre-existing dyspareunia as a risk factor for sexual health issues 6 and 12 months postpartum

	6 months postpartum					12 months postpartum				
	n/total	%	Unadjusted OR	95% CI	*p* value	n/total	%	Unadjusted OR	95% CI	*p* value
Dyspareunia	126/234	53.8	2.5	1.8–3.5	< 0.001*	85/235	36.2	3.2	2.3–4.6	< 0.001*
Lack of vaginal lubrication	123/242	50.8	1.6	1.1–2.1	0.004*	106/237	44.7	1.8	1.3–2.5	< 0.001*
Loss of interest in sexual activity	127/242	52.5	1.4	1.0–1.9	0.025*	110/237	46.4	1.5	1.1–2.0	0.01*

*indicates statistical signficance at *p* < 0.05

### Multivariable logistic regression analysis

#### Dyspareunia at 6 and 12 months postpartum

Pre-existing dyspareunia was strongly associated with dyspareunia (over two and half times more likely) at 6 months postpartum (Adjusted OR (AOR) 2.6, 95% CI 1.8–3.6), and this association was even more pronounced at 12 months (AOR 3.8, 95% CI 2.5–5.8). Breastfeeding and a 3rd degree perineal tear were both associated with experiencing dyspareunia 6 months after birth. Having a vacuum-assisted birth was not a significantly associated risk factor for dyspareunia 6 months postpartum (AOR 1.7, 95% CI 0.9–2.7). Compared to women aged 18–29 years, women aged ≥30 years were less likely to experience dyspareunia at 6 and 12 months. This was most pronounced at 12 months for women ≥35 years of age (Table 7).

**Table 7:** Multivariable logistic regression of dyspareunia at 6 and 12 months postpartum

Associated factors		6/12 postpartum			12/12 postpartum		
		Total *n* = 748			Total *n* = 585		
		OR	95% CI	*p* value	OR	95% CI	*p* value
Age Groups	18–29 years	1.0 (ref.)			1.0 (ref.)		
	30–34 years	0.7	0.4–1.0	0.059	0.7	0.4–1.2	0.222
	35+ years	0.7	0.4–1.0	0.096	0.4	0.2–0.8	0.009*
BMI Groups	Ideal	1.0 (ref.)			1.0 (ref.)		
	Overweight	1.0	0.6–1.6	0.947	0.9	0.5–1.7	0.773
	Obese	1.1	0.6–1.8	0.841	0.8	0.4–1.7	0.545
	Underweight	1.4	0.7–2.7	0.325	1.4	0.6–3.4	0.387
	Unknown BMI	0.7	0.3–1.4	0.366	0.9	0.4–2.5	0.926
Highest level of education	No degree	1.0 (ref.)			1.0 (ref.)		
	Primary degree	1.4	0.9–2.1	0.11	1.3	0.7–2.3	0.35
	Postgrad qualification	1.1	0.7–1.6	0.698	1.3	0.8–2.3	0.283
Pre-existing dyspareunia	Yes	2.6	1.8–3.6	< 0.001*	3.8	2.5–5.8	< 0.001*
Mode of birth	SVB	1.0 (ref.)			1.0 (ref.)		
	Vacuum birth	1.7	0.9–2.7	0.053	1.5	0.7–2.8	0.225
	Forceps birth	0.7	0.3–1.4	0.384	0.8	0.3–1.8	0.611
	Elective CS	0.7	0.3–1.7	0.491	1.9	0.6–5.7	0.255
	Emergency CS	1.1	0.6–2.2	0.605	1.537	0.6–3.7	0.344
Perineal trauma	Intact^a^	1.0 (ref.)			1.0 (ref.)		
	2nd degree	1.6	0.8–3.1	0.133	1.4	0.5–3.4	0.466
	3rd degree	4.1	1.3–12.3	0.013*	2.7	0.7–10.1	0.143
	Episiotomy	1.4	0.7–2.7	0.336	1.5	0.6–3.6	0.374
Still breastfeeding	Yes	1.9	1.3–2.7	< 0.001*	1.1	0.7–1.9	0.56
Perception of body image	Always satisfied	1.0 (ref.)			1.0 (ref.)		0.993
	Sometimes satisfied	0.9	0.6–1.5	0.96	1.0	0.6–1.7	0.941
	Never satisfied	1.4	0.8–2.4	0.211	1.0	0.5–2.2	0.905

^a^includes 1st degree tears and vaginal wall and labial tears

*indicates statistical signficance at *p* < 0.05

#### Lack of vaginal lubrication at 6 and 12 months postpartum

Pre-existing dyspareunia was strongly associated with a lack of vaginal lubrication at 6 months (AOR 1.6, 95% CI 1.1–2.2) and the association persisted to 12 months postpartum (AOR 1.7, 95% CI 1.2–2.5). Breastfeeding, being sometimes satisfied with one’s body image and never satisfied with one’s body image were all associated with a lack of vaginal lubrication 6 months postpartum. Compared to ideal weight women, being overweight or obese was protective of experiencing a lack of vaginal lubrication 6 months after birth. A non-significant association between a vacuum-assisted birth and an increased lack of vaginal lubrication at 12 months was also found (Table 8).

**Table 8:** Multivariable logistic regression of lack of vaginal lubrication at 6 and 12 months postpartum

Associated factors		6/12 postpartum			12/12 postpartum		
		Total *n* = 758			Total *n* = 591		
		OR	95% CI	*p* value	OR	95% CI	*p* value
Age Groups	18–29 years	1.0 (ref.)			1.0 (ref.)		
	30–34 years	0.9	0.7–1.4	0.994	0.8	0.5–1.3	0.383
	35+ years	0.9	0.6–1.4	0.799	0.7	0.4–1.1	0.149
BMI Groups	Ideal	1.0 (ref.)			1.0 (ref.)		
	Overweight	0.5	0.3–0.8	0.003*	0.7	0.3–1.1	0.129
	Obese	0.5	0.3–1.0	0.038*	0.6	0.3–1.2	0.148
	Underweight	1.5	0.8–2.9	0.226	1.8	0.9–3.7	0.117
	Unknown BMI	0.6	0.3–1.2	0.182	0.9	0.4–2.0	0.896
Highest level of education	No degree	1.0 (ref.)			1.0 (ref.)		
	Primary degree	1.0	0.6–1.5	0.985	1.0	0.6–1.7	0.862
	Postgrad qualification	1.1	0.8–1.7	0.496	1.2	0.8–2.0	0.332
Pre-existing dyspareunia	Yes	1.6	1.1–2.2	0.005*	1.7	1.2–2.5	0.004*
Mode of birth	SVB	1.0 (ref.)			1.0 (ref.)		
	Vacuum birth	1.4	0.9–2.4	0.145	1.7	1.0–3.0	0.062
	Forceps birth	1.0	0.6–1.9	0.932	1.4	0.7–2.9	0.308
	Elective CS	0.7	0.3–1.5	0.339	1.2	0.5–2.9	0.677
	Emergency CS	0.9	0.5–1.7	0.874	1.3	0.6–2.7	0.405
Perineal trauma	Intact^a^	1.0 (ref.)			1.0 (ref.)		
	2nd degree	1.0	0.5–1.8	0.963	1.0	0.5–2.0	0.945
	3rd degree	1.4	0.5–3.7	0.55	1.2	0.4–3.8	0.787
	Episiotomy	0.8	0.4–1.5	0.449	0.7	0.3–1.4	0.325
Still breastfeeding	Yes	2.1	1.5–2.9	< 0.001*	1.3	0.8–1.9	0.27
Perception of body image	Always satisfied	1.0 (ref.)			1.0 (ref.)		
	Sometimes satisfied	1.8	1.2–2.8	0.005*	1.2	0.7–1.9	0.444
	Never satisfied	2.4	1.4–4.0	0.001*	1.5	0.8–2.8	0.233

^a^includes 1st degree tears and vaginal wall and labial tears

*indicates statistical signficance at *p* < 0.05

#### Loss of interest in sexual activity at 6 and 12 months postpartum

Breastfeeding at 6 months and 12 months postpartum were associated with experiencing a loss of interest in sexual activity at these time-points (AOR 2.2, 95% CI 1.6–3.0 and AOR 1.6, 95% CI 1.0–2.1, respectively). Being sometimes satisfied and never satisfied with one’s body image was a risk factor for a loss of interest in sexual activity 6 months after birth. This association persisted for women who were never satisfied with their body image to 12 months postpartum (AOR 3.6, 95% CI 1.9–6.7). Compared with women without degree-level educational qualifications, women who had a postgraduate qualification were more likely to experience a loss of interest in sexual activity 6 months after birth (AOR 1.5, 95% CI 1.0–2.3) (Table 9).

**Table 9:** Multivariable logistic regression of loss of interest in sexual activity at 6 and 12 months postpartum

Associated factors		6/12 postpartum			12/12 postpartum		
		Total *n* = 762			Total *n* = 588		
		OR	95% CI	*p* value	OR	95% CI	*p* value
Age Groups	18–29 years	1.0(ref.)			1.0(ref.)		
	30–34 years	0.8	0.6–1.2	0.396	1.0	0.7–1.6	0.836
	35+ years	0.8	0.5–1.3	0.436	1.2	0.7–2.0	0.441
BMI Groups	Ideal	1.0(ref.)			1.0(ref.)		
	Overweight	0.9	0.6–1.4	0.562	0.7	0.4–1.3	0.276
	Obese	1.6	1.0–2.8	0.07	0.8	0.4–1.5	0.42
	Underweight	0.9	0.5–1.9	0.982	0.9	0.4–2.0	0.882
	Unknown BMI	0.7	0.4–1.4	0.337	0.9	0.4–2.1	0.931
Highest level of education	No degree	1.0(ref.)			1.0(ref.)		
	Primary degree	1.0	0.7–1.5	0.916	1.3	0.8–2.0	0.326
	Postgrad qualification	1.5	1.0–2.3	0.021*	1.3	0.8–2.0	0.249
Pre-existing dyspareunia	Yes	1.4	1.0–1.9	0.05*	1.3	0.9–1.9	0.127
Mode of birth	SVB	1.0(ref.)			1.0(ref.)		
	Vacuum birth	1.1	0.7–1.8	0.686	1.4	0.8–2.4	0.235
	Forceps birth	0.7	0.4–1.3	0.244	1.3	0.6–2.5	0.489
	Elective CS	0.7	0.4–1.6	0.446	1.0	0.4–2.2	0.933
	Emergency CS	0.8	0.4–1.5	0.464	0.7	0.3–1.3	0.266
Perineal trauma	Intact^a^	1.0(ref.)			1.0(ref.)		
	2nd degree	1.6	0.9–2.9	0.125	1.0	0.5–2.0	0.941
	3rd degree	2.6	0.9–7.2	0.065	0.9	0.3–2.8	0.853
	Episiotomy	1.2	0.6–2.3	0.595	0.7	0.3–1.4	0.272
Still breastfeeding	Yes	2.2	1.6–3.0	< 0.001*	1.6	1.0–2.4	0.029*
Perception of body image	Always satisfied	1.0(ref.)			1.0(ref.)		
	Sometimes satisfied	1.6	1.0–2.4	0.035*	1.5	0.9–2.3	0.082
	Never satisfied	2.8	1.6–4.6	< 0.001*	3.6	1.9–6.7	< 0.001*

^a^includes 1st degree tears and vaginal wall and labial tears

*indicates statistical signficance at *p* < 0.05

## Discussion

Discourse on women’s sexual health after birth is gaining momentum across diverse disciplines, for example, midwifery, obstetric, sexology and psychology disciplines [1–5]. This increased interest and body of research in perinatal sexual health, however, is not evidenced in sexual health policy [6, 7] or maternity care policy [8, 9], although data demonstrating that women are not prepared for changes to their sexual health after birth [10], are available. Lack of knowledge and preparation for sexual health issues postpartum can be distressing for women, and their partner, while also negatively impacting on their ability to adapt to their new role as mothers [10–12]. Postpartum sexual health is challenging to theoretically define but cannot be separated from sexuality and sexual function, and is thought to be influenced by labour and birth events [13]. Attributes of good postpartum sexual health include; sexual desire, resumption of sexual intercourse after birth, pain free sex and orgasm.

### Key findings

This study provides a further body of evidence demonstrating that women experience considerable sexual health issues after pregnancy and childbirth, and adds to the discourse on women’s sexual health after birth from a maternity (midwifery and obstetric) perspective. Almost half of the women included in this study reported sexual health issues 6 months postpartum with more than 40% doing so 12 months after birth. A loss of interest in sexual activity was the most commonly reported issue (46.3% at 6 months and 39.8% at 12 months). This is somewhat less than that reported in the Australian Maternal Health Study (60.3% at 6 and 51.3% at 12 months) [1] and more than that reported by Barrett and colleagues at 6 months postpartum (37%) [17]. Information relating to sexual health issues that was sought in these 3 studies were almost identical; however, there is a 15-year interval from data collection in our study and that of Barrett and colleagues. It is, therefore, possible that over the past 15 years women have become more comfortable and confident in recognising sexual health issues, possibly as a result of the increased interest in the social media, weekender magazines and in other media which discuss women’s sexual lives after birth [31, 32]. Experiencing a loss of interest in sexual activity during the first year after birth is relatively common, which suggests that altered desire for sex is a normal part of adapting to motherhood and new roles of both parents in the household. If viewed through the adaptation lens, one is left with questions around the appropriateness of including lack of sexual activity as an indicator of ‘sexual dysfunction’ in the DSM-5 definition of sexual dysfunctions [33], especially for this cohort of postpartum women. The high rate of reported loss of interest in sexual activity also points to the need for women and their partners to be forewarned of this potential change, as a routine part of perinatal care. By so doing much of the stress and anxiety identified by women interviewed by Olsson [10] and guilt reported by women in Woolhouse and colleague’s study [11] around intimacy would be reduced.

In our study 37.5% of women experienced dyspareunia 6 months after birth, compared to 43.4% reported in the Maternal Health Study [1] and 31% in Barrett et al.’s (2000) study [17]. Our findings demonstrate that events that occur during labour and birth influence the extent with which women report dyspareunia 6 months after birth. The likelihood of women experiencing dyspareunia at 6 months was substantially higher in women whose birth was vacuum-assisted, had 2nd degree tears, 3rd degree tears and episiotomies compared to those who had a spontaneous vaginal birth and an intact perineum; although, when all other factors were considered, 3rd degree tears, only, along with pre-existing dyspareunia and breastfeeding emerged as significant factors for dyspareunia at 6 months postpartum. Our univariate results reflect the findings from previous studies which also report an association with episiotomy and poor sexual health outcomes [15], instrumental birth and dyspareunia [19, 34]. In addition, it raises questions about the rates of obstetric intervention experienced by women in Ireland. In our study 20.7% of women experienced a vacuum-assisted birth, similar to a national rate of 21.2% [29], double the rate of 10.4% in the Maternal Health Study in Australia [34] and much higher than the 5% in the nulliparous sample used by Connolly and colleagues [35]. Our high rate of vacuum-assisted birth could be related to the equally high uptake of epidural anaesthesia in Irish maternity settings, as 78% of women in this study used epidural analgesia (similar to the 72% of nulliparous women at the research site), and a 2011 Cochrane review identified that epidural analgesia increased the risk of an instrumental birth [36]. The association between episiotomy and persistent dyspareunia up to 12 months was found in our study, although it did not emerge as a risk factor for dyspareunia in multivariable analysis. In our study 36.1% of women had an episiotomy, while this may appear elevated it is worth noting that 33% of women had an instrumental birth which is commonly associated with an episiotomy. Our high rate of episiotomy (36.1%) compares poorly, internationally, where 16% of women in the Maternal Health Study had an episiotomy [34] and 14% in Connolly’s research [35]. This finding does not necessarily suggest there is routine use of episiotomy but rather poses concern over the high rate of epidural uptake, consequent instrumental births, perineal trauma and associated long term dyspareunia.

Little has been published on the influence of breastfeeding on postpartum sexual health, with many studies choosing to focus on breastfeeding as a means of contraception [37] or the influence of breastfeeding on resumption of sexual activity and frequency of sexual activity [38–40]. In our study, breastfeeding, in association with other related factors, remained significantly present for all three of the outcomes of dyspareunia, a lack of vaginal lubrication and a loss of interest in sexual activity 6 months postpartum. This finding highlights the potential for cognitive dissonance to occur. Cognitive dissonance occurs when people experience inconsistency between cognitions or between cognitions and behaviour [41]. In a professional or practice context that emphasises women-centred care and disclosure, and a policy context that promotes breastfeeding, there is potential for internal conflict to arise. Practitioners may struggle with the professional imperative to inform women of the impact of breastfeeding on sexual activity, dyspareunia and vaginal lubrication at the same time as fearing a decrease in women’s willingness to breastfeed if impact is known. However, information regarding breastfeeding needs to take account of these findings, if care is to be ‘woman-centred’ as opposed to ‘breast-feeding centred’ [42]. Without this information women may blame themselves for their loss of sexual interest, or struggle alone without information on the array of vaginal lubricants available to alleviate vaginal dryness.

Little attention has been given to pre-existing dyspareunia and its influence on sexual health after birth to date, however two studies found a similar association between pre-existing dyspareunia and experiencing sexual health issues after birth [17, 34]. In our study 29.3% of women experienced dyspareunia in the 12 months before becoming pregnant, and this, with other significantly related factors (e.g. 3rd degree tears and breastfeeding at 6 months and age > 35 years at 12 months) contributed to dyspareunia 6 and 12 months after birth. The majority of women do not seek professional support for postpartum sexual health issues, 15% in Barrett et al.’s study spoke to a health professional [17] and 24% in the Australian study were asked directly by a health professional about their sexual health postpartum [1]. This corresponds to conclusions from qualitative studies that demonstrated that women find it difficult to bring up sexual health issues with health professionals [10, 11, 43] and this occurs at a time when women have direct contact with a variety of health professionals during the postpartum period. Therefore it is very likely that women do not seek help for dyspareunia experienced before pregnancy as there may be limited contact with health services. The antenatal period, a time when women have frequent consultations with health professionals appears to be an ideal opportunity to ask them about their sexual health and discuss any problems, such as pain during sexual intercourse, they may be experiencing. It is potentially an ideal time to refer women to the most appropriate professional for help, be it the women’s health physiotherapist attached to the maternity services, sexual health therapist or couples therapy. However, previous studies of healthcare professionals have shown that many lack competence and confidence in their abilities to help with sexual problems [44], which may be why so many women had not been asked. Managing dyspareunia during pregnancy will go some way to reducing the identified association between pre-pregnancy dyspareunia and a lack of vaginal lubrication and a loss of interest in sexual activity seen in this study. Similarly, it is probable that persistent postpartum dyspareunia at 6 and 12 months would be reduced if managed antenatally or at the very least women should be asked about sexual health issues, and would then know where to seek appropriate help.

This study is unique in its investigation of an association between perception of body image and sexual health issues after birth. In this study women with a poor perception of their body images 6 and 12 months postpartum were more likely to experience a lack of vaginal lubrication (in the context of being overweight, obese, breastfeeding and pre-existing dyspareunia) and a loss of interest in sexual activity (in the context of breastfeeding and pre-existing dyspareunia). The complex nature of the concept of postpartum body image and its influence on postpartum sexual health is poorly researched, and this led the first author of this paper to carry out qualitative one-to-one semi-structured interviews with some of the women who completed the survey and identified themselves as experiencing sexual health problems. Analysis of these data is in progress and will be reported at a later date.

### Strengths and limitations

The strengths of this study include the recruitment of a large sample of nulliparous women in early pregnancy, regular follow-up and a high retention rate to 12 months postpartum. The frequency of follow-up reduces the likelihood of recall bias and provides reliable data on changes to women’s sexual health over time following birth. Some findings in our study are similar to other comparable studies. This strengthens the argument for introducing sexual health to antenatal and postnatal care pathways well beyond the traditional 6 week postnatal assessment.

A number of potential limitations have been identified that may influence the data. The study sample is from one maternity unit in Ireland, which is not entirely representative of a national sample. The survey did not include definitions of concepts such as lack of vaginal lubrication, hence they are open to individual interpretation on meaning. The association of breastfeeding and sexual health issues may be questionable as Ireland has a low breastfeeding continuation rate; for example, in a national study of infant feeding in Ireland, only 19% (*n* = 347) of women were exclusively breastfeeding at 3–4 months postpartum [45]. Data on other factors such as medications (e.g., psychotropic drugs) that may affect interest in sexual activity [46] were not collected. A further limitation is the lack of data on the sexual orientation of women in our study, thus it was not possible to identify if there was any difference between women in same sex relationships and those in opposite sex relationships.

## Conclusion

The findings from this large prospective cohort study of nulliparous demonstrates that women experience considerable sexual health issues after pregnancy and childbirth. Dyspareunia, lack of vaginal lubrication and loss of interest in sexual activity at 6 months postpartum were all significantly associated with pre-existing dyspareunia and breastfeeding. Additionally dyspareunia was associated with 3rd degree tears, lack of vaginal lubrication was associated with being overweight, obese and dissatisfaction with body image was a risk factor for a lack of vaginal lubrication and a loss of interest in sexual activity. Preparing women and their partners for this during the antenatal period and advising on simple measures, such as use of lubrication to avoid issues, could potentially remove stress, anxiety and fears regarding intimacy after birth. Pregnancy and the frequent interactions it brings with health professionals provide an ideal opportunity to discuss pre-existing sexual health issues with women and their partners and suitable care pathways can be put in place with appropriate referrals made.

## Abbreviations

AOR: Adjusted Odds Ratio
BMI: Body Mass Index
CI: Confidence Interval
CS: Caesarean Section
MAMMI: Maternal health And Maternal Morbidity in Ireland
OR: Odds ratio
PP: Postpartum
SPSS: Statistical Package for the Social Sciences
